# The DISC (Diabetes in Social Context) Study-evaluation of a culturally sensitive social network intervention for diabetic patients in lower socioeconomic groups: a study protocol

**DOI:** 10.1186/1471-2458-12-199

**Published:** 2012-03-19

**Authors:** Charlotte Vissenberg, Vera Nierkens, Paul JM Uitewaal, Diana Geraci, Barend JC Middelkoop, Giel Nijpels, Karien Stronks

**Affiliations:** 1 Department of Public Health, Academic Medical Center, University of Amsterdam, Amsterdam, The Netherlands; 2 The Hague's Public Health Department, The Hague, The Netherlands; 3 Leiden University Medical Centre, Leiden, The Netherlands; 4 VU University Medical Centre, Amsterdam, The Netherlands

**Keywords:** Type 2 diabetes, Lower socioeconomic groups, Turkish, Moroccan, Surinamese patients, Social network intervention, Diabetes self-management

## Abstract

**Background:**

Compared to those in higher socioeconomic groups, diabetic patients in lower socioeconomic groups have less favourable metabolic control and experience more diabetes-related complications. They encounter specific barriers that hinder optimal diabetes self-management, including a lack of social support and other psychosocial mechanisms in their immediate social environments. *Powerful Together with Diabetes *is a culturally sensitive social network intervention specifically targeted to ethnic Dutch, Moroccan, Turkish, and Surinamese diabetic patients in lower socioeconomic groups. For ten months, patients will participate in peer support groups in which they will share experiences, support each other in maintaining healthy lifestyles, and learn skills to resist social pressure. At the same time, their significant others will also receive an intervention, aimed at maximizing support for and minimizing the negative social influences on diabetes self-management. This study aims to test the effectiveness of *Powerful Together with Diabetes*.

**Methods/Design:**

We will use a quasi-experimental design with an intervention group (Group 1) and two comparison groups (Groups 2 and 3), N = 128 in each group. Group 1 will receive *Powerful Together with Diabetes*. Group 2 will receive *Know your Sugar*, a six-week group intervention that does not focus on the participants' social environments. Group 3 receives standard care only. Participants in Groups 1 and 2 will be interviewed and physically examined at baseline, 3, 10, and 16 months. We will compare their haemoglobin A1C levels with the haemoglobin A1C levels of Group 3. Main outcome measures are haemoglobin A1C, diabetes-related quality of life, diabetes self-management, health-related, and intermediate outcome measures. We will conduct a process evaluation and a qualitative study to gain more insights into the intervention fidelity, feasibility, and changes in the psychosocial mechanism in the participants' immediate social environments.

**Discussion:**

With this study, we will assess the feasibility and effectiveness of a culturally sensitive social network intervention for lower socioeconomic groups. Furthermore, we will study how to enable these patients to optimally manage their diabetes. This trial is registered in the Dutch Trial Register: NTR1886

## Background

Lower socioeconomic (SE) groups are not only disproportionately affected by type 2 diabetes, they also have more diabetes-related complications and higher diabetes-related mortality compared to diabetic patients in higher SE groups [1-3]. A possible explanation for this could be poorer glycaemic control. Achieving optimal glycaemic control requires the diabetic patient to take part in a complex set of tasks: adhere to dietary advice and medications, engage in regular physical activity, quit smoking, and monitor blood glucose levels, known as diabetes self-management (DSM) [4]. These tasks seem to be more challenging for diabetic patients in lower SE groups [5-7].

Interventions aimed at improving DSM can contribute to better glycaemic control and the prevention of diabetes-related complications [8,9]. However, there are indications that interventions for the general diabetic population are less suitable for lower SE groups and need to be adapted to the specific barriers they face [10,11]. Barriers to DSM among lower SE groups include a lack of knowledge of diabetes, low self-efficacy, low perceived control, and low health literacy [5,7,12].

Another mentioned barrier to DSM among lower SE groups is a lack of diabetes-related social support [13-15]. To maintain lifestyle changes, long-term social support in particular seems beneficial [13,16]. From other fields such as sociology we know that social support is not the only psychosocial mechanism through which the immediate social environment influences health [17-19]. Other psychosocial mechanisms are social influence such as peer pressure, norms and social comparison processes, which extend from the social network's values and norms, and social engagement which defines and reinforces meaningful social roles through network participation [20]. These psychosocial mechanisms may have both negative and positive effects on health behaviour [17]. For example, if the social norms in the immediate social environment are incongruent with DSM, family and/or friends might hinder DSM (intentionally or unintentionally) by providing negative role models or by exerting peer pressure [17,20,21].

Patients in lower SE groups are often surrounded by social networks that seem to have a strong adverse effect on their DSM. These groups often have small, dense social networks with strong ties that consist primarily of people in similar (low SE) situations to their own, mainly family members and close friends [18]. Their social networks often lack more distant acquaintances, that is, people who move in different circles than their own. These distant acquaintances are needed to introduce resources, information, and ideas that might be missing from their own social networks (such as diabetes related information and role models). These 'closed' social networks are known to impose strong group norms on their social network members [18,20]. Combined with the unfavourable diabetes outcomes among lower SE groups, this may suggest that these groups are confronted with strong psychosocial mechanisms (social support, social influence and social engagement) in their immediate social environments that adversely affect their DSM. This is supported by recent studies which show that lower SE groups experience difficulties in coping effectively with psychosocial mechanisms such as peer pressure, cultural expectations, fewer positive role models, and not having control over their family's lifestyle choices [22-24].

To improve DSM among lower SE groups, it seems important to focus specifically on creating long-term diabetes-related social support, and to target the other existing psychosocial mechanisms such as social influence and social engagement in their immediate social environments that have adverse effects on their DSM. Therefore, we systematically developed a theory-based culturally sensitive social network intervention targeted to ethnic Dutch, Turkish, Moroccan, and Surinamese diabetic patients in lower SE groups. *Powerful Together with Diabetes *(PTWD) aims to improve DSM by stimulating long-term social support and targeting existing psychosocial mechanisms in the immediate social environment that have an adverse effect on DSM. This paper describes the intervention, the theoretical background of this intervention, and the study design.

## Methods/Design

### The intervention: *Powerful Together with Diabetes (PTWD)*

To develop PTWD, we used the intervention mapping method: a systematic approach for designing and evaluating health-promotion interventions. The first step in intervention mapping is a needs assessment, followed by creating matrices of change objectives, selecting theory-informed intervention methods and practical strategies, and finally, producing programme components and materials [25].

For the needs assessment, we examined the existing literature for barriers to DSM among lower SE groups. We also held semi-structured qualitative interviews with diabetic patients (n = 21), analysed the content of an internet forum for diabetic patients, and observed the daily practices of a diabetic nurse in a neighbourhood with low socioeconomic status for two days. We created matrices of change objectives with the help of researchers specialized in intervention mapping. When selecting theory-informed intervention methods and practical strategies and producing programme components and materials, we examined the existing literature and currently ongoing lifestyle interventions for methods and strategies that would fit our target population. We also submitted our practical strategies and programme components twice to a panel of experts with Turkish, Moroccan, and Surinamese backgrounds (n = 6), and consulted them individually with specific questions about the different cultural groups in our target population. Finally, we pretested some of our intervention components among the target population using focus group discussions (n = 3).

Because this intervention has to stimulate long-term social support and target existing psychosocial mechanisms in DSM, PTWD will consist of the following three components: 1) group meetings for diabetic patients, 2) group meetings for the participants' significant others (e.g. family and/or friends), and 3) social network therapy sessions with the diabetic patient and his/her significant other(s). PTWD will last ten months and consist of two phases (see Table 1).

**Table 1:** Overview of the two phases of *Powerful Together with Diabetes*

Phase 1 (months 1-3)	Phase 2 (months 4-10)
Thirteen weekly peer group meetings for diabetic patients. Three group meetings for significant others.	Eight twice-monthly peer group meetings for diabetic patients followed by five monthly peer group meetings. Three group meetings for significant others. Two social network therapy sessions for the diabetic patient and his/her significant other(s).

The intervention will be held in the mother tongue of the participants. We culturally targeted PTWD by changing the outline of the intervention to make it compatible with the ethnic minority participants' annual visits to their countries of origin and the celebration of Ramadan. We also culturally tailored the content of the intervention components to the different cultural groups, for example, by incorporating sociocultural values and barriers to DSM, and adapting the materials to fit the needs of the different cultural groups.

We describe the intervention components below, including their behavioural goals and the determinants we addressed to achieve these goals. The behavioural goals were based on the theory of self-regulation, different self-management theories, and the transactional model of stress and coping, relapse prevention, and social learning theories [26-30]. Because targeting psychosocial mechanisms within the immediate social environment of the participants (such as the exchange of social support) plays an important part in this intervention, for every intervention component we also created separate behavioural goals for these determinants.

#### Group meetings for diabetic patients

The diabetic patients will participate in group meetings (ten persons per group) for ten months. In Phase 1 (months 1-3), the participants will be provided with the basic 'tools' they need to manage their diabetes. This phase focuses on creating positive outcome expectations [26,31] and moral norms [28,32], increasing knowledge, skills [26], and self-efficacy [26,31], stimulating social support [33], and recognizing and dealing with psychosocial mechanisms that hinder optimal DSM, such as peer pressure and existing social norms [20,31]. In Phase 2 (months 4-10), the participants will learn how to put the tools they gathered in Phase 1 to use. They will develop and train DSM skills until they have a solid set of coping skills [28,29,34], which will enable them to optimally manage their diabetes in the long term.

To reach these behavioural goals and change the determinants associated with them, we chose strategies that would fit our target population and their specific needs regarding information processing. For example, by engaging the participants in an interactive diabetes quiz, we aim to increase their practical knowledge about diabetes. We aim to increase DSM skills and self-efficacy through a 'letter of the week', in which a fictitious diabetic patient presents a problem and asks the group members for advice. To increase awareness about barriers to DSM (Phase 2), the participants will monitor their own behaviour with the help of specially designed diaries. Through participatory problem-solving and rehearsal situations, we aim to improve the participants' skills and self-efficacy to overcome these barriers. Due to the large number of behavioural goals, in Additional files 1 and 2 we present a representative summary of the behavioural goals, the determinants addressed, and the strategies used in Phases 1 and 2.

Stimulating long-term social support and targeting existing psychosocial mechanisms that have an adverse effect on DSM are important parts of the intervention. Examples of strategies used to stimulate social support are interactive games in which the participants complement each other on their DSM and other subjects (self-affirmation), and stimulating the exchange of advice between participants. The participants are also encouraged to see each other outside of the group meetings, and to undertake DSM-related activities together (e.g. participating in an exercise initiative in the neighbourhood). A final aim of the intervention is that the participants will continue to see and consult each other after the intervention has ended.

To stimulate social support and target existing psychosocial mechanisms that have an adverse effect on DSM in participants' immediate social environments, we use role-model stories, rehearsal situations, and homework assignments that focus on these behavioural goals. In addition, the group meetings for the significant others and the social network therapy sessions (see below) will contribute to the achievement of this behavioural goal.

#### Group meetings for significant others

Each diabetic patient will identify one or two persons in his/her immediate environment with a great deal of influence on his/her DSM. These significant others will participate in six group meetings with other significant others: three during Phase 1 and three during Phase 2. The diabetic patients will not be present during these meetings.

In Phase 1, we will aim to increase the significant others' knowledge about diabetes and its treatment, make them feel that DSM is necessary and inevitable (outcome expectations and perceived cultural norms), and that they have an important role in the DSM of their relative/friend (perceived cultural norms and self-efficacy). To increase the significant others' knowledge, we will use a short version of the interactive diabetes quiz (which we use in the group meetings for the diabetic patients). Using positive role-model stories and shared positive experiences from group members (vicarious reinforcement) and group discussions, we will try to increase positive outcome expectations and positive moral norms towards DSM, and increase awareness about the role of significant others in the DSM of diabetic patients.

In Phase 2, we will focus on supporting their relative/friend in managing his/her diabetes. We will aim to make the significant others aware of the fact that DSM is a shared responsibility. Next, we will aim to make them feel confident they can support the diabetic patient with his/her DSM (self-efficacy) and be able to effectively support the diabetic patient in his/her DSM (skills). By exchanging positive experiences and through communication skills training combined with rehearsal situations and feedback, we will aim to improve the significant others' skills and self-efficacy (see Additional file 3). The group meetings will be followed by the social network therapy sessions.

#### Social network therapy sessions

In Phase 2, the diabetic patient and his/her significant other(s) will take part in two social network therapy sessions. With these sessions, we will aim to further stimulate the exchange of social support and diminish negative social influences on DSM. In these sessions, the group leader will lead a discussion between the diabetic patient and his/her significant other(s). In the first meeting, they will discuss the ways the diabetic patient is currently managing his/her diabetes and how his/her DSM could be improved. Next, they will discuss the ways the significant other(s) can assist the diabetic patient in improving his/her DSM. Finally, they will make an action plan to put the things discussed into action. In the second meeting, this action plan will be evaluated and further refined (see Additional file 4).

### Intervention for the comparison group: *Know your Sugar*

To evaluate the effects of an extra focus on psychosocial mechanisms and social support in the intervention group, we chose to offer the comparison group a lifestyle group intervention as well. *Know your Sugar *(KYS) is a group intervention that aims to provide the participants with the information they need to be able to manage their diabetes, and provide them with the opportunity to exchange social support. KYS will not actively focus on social support and the pre-existing psychosocial mechanisms that influence DSM in the participants' immediate social environments.

KYS consists of six weekly group meetings. It is based on 'How to deal with diabetes', a Dutch course originally developed for Turkish diabetic patients, and Dutch National Institute for Health Promotion and Disease Prevention (NIGZ) posters (i.e. large visual aids used by diabetic nurses to facilitate explaining and talking about diabetes-related subjects with patients). Both are known as the current best practices in the Netherlands for diabetic patients in lower SE groups. Together they form the foundation of KYS, which we then further developed to make it culturally sensitive for the different cultural groups in our study population. To increase the participants' knowledge, we will use the same strategies we use in PTWD, but with no extra focus on the exchange of experiences and social support.

#### Training and supervision of group leaders

PTWD and KYS will be given by different group leaders. The PTWD group leaders will receive a four-hour training prior to Phase 1 and a four-hour training prior to Phase 2. The KYS group leaders will receive a two-hour training prior to the intervention. During the interventions, all group leaders will have regular contact with the researchers by telephone. During these calls they can ask questions about the intervention and get practical advice.

### Research questions

This study will aim to answer the following research questions:

1. What are the effects of the intervention (PTWD) on haemoglobin A1C (HbA1c) compared with the intervention for the comparison group (KYS) and standard care at 3, 10, and 16 months?

2. What are the effects of the intervention on diabetes-related quality of life compared with the intervention for the comparison group at 3, 10, and 16 months?

3. What are the effects of the intervention on health-related outcome measures, diabetes self-management, and intermediate outcome measures compared with the intervention for the comparison group at 3, 10, and 16 months?

4. What is the feasibility of the intervention and the intervention for the comparison group?

5. What is the cost-effectiveness of the intervention compared with the intervention for the comparison group and standard care?

### Study design

The intervention effects will be measured in a quasi-experimental controlled trial: the DISC (Diabetes in Social Context) Study. The participants who receive the intervention (PTWD, N = 128) will be compared with the participants of the comparison group (KYS, N = 128). In addition, the HbA1c levels of the participants in both groups will be compared with the HbA1c levels of 128 diabetic patients who will receive no intervention at all (standard care).

### Matching, blinding, recruitment, and informed consent

For the intervention to be successful, it is important that the participants live near each other. Therefore randomization is impossible. Therefore, the intervention and comparison group will be matched according to ethnicity, gender, and organization of diabetes care. Blinding the general practitioners (GPs) is impossible due to the nature of this study.

Recruiting people and maintaining participation in lower SE groups in intervention studies is difficult due to a low level of trust and insufficient understanding of the study and study procedures [35-38]. To increase trust and understanding, the eligible patients will be invited to a 'welcome meeting' about PTWD or KYS by their GP, a person they trust and respect. For people who do not speak Dutch, we will organize meetings in their mother tongue. At the welcome meetings, the eligible patients will receive detailed information about the study procedures. They will also take part in one of the intervention components (in PTWD groups, a short version of the game about nutrition, in KYS groups a short version of the diabetes quiz) to get an idea of what the intervention will be like. They will also meet their group leaders and the other patients and will see the intervention location, which we hope will lower barriers to participation.

At the end of the welcome meeting, the participants have two weeks to consider their participation. After two weeks they will be asked to sign an informed consent form in which they agree to participate in the intervention and give their consent for the study procedures. The informed consent form will be read to the diabetic patient and further explained if necessary. People who do not speak Dutch will receive the information in their mother tongue.

### Ethical considerations

This study has been approved by the Medical Ethics Committee of the Academic Medical Center (AMC) in Amsterdam, and is registered with the Dutch Trial Register (Dutch Trial Register NTR1886).

### Study population

We will include people with type 2 diabetes who received medical treatment long enough to achieve optimal glycaemic control (one year) but still have a HbA1c above 7% [39]. Inclusion criteria are at least one year since diagnosis, HbA1c > 7%, > 35 years, and living in a neighbourhood with low socioeconomic status. Exclusion criteria are GP objection to participation, severe psychiatric disorders, being unable to come to the intervention location independently, and planning to stay abroad for longer than six weeks during the intervention period.

Taking into account the prevalence of type 2 diabetes among ethnic minorities in lower SE groups in the Netherlands, we expect half of the study population to consist of ethnic Dutch patients and the other half to come from minority groups, in particular patients with Turkish, Moroccan, and Surinamese backgrounds.

### Sample size calculation

The primary outcome measure is the decrease in HbA1c at 16 months. A decrease of 0.5% in the intervention group compared to the comparison group will be seen as a difference that is clinically relevant. This means that at a statistical significance of 5% and a power of 80%, we will need 92 participants in both the intervention and the comparison group. Bearing in mind a dropout rate of 40%, we will need to include 128 people in both the intervention group and the comparison group.

### Measurements

#### Primary and secondary outcome measures

The primary outcome measures are the HbA1c levels of the participants and diabetes-related quality of life at 16 months after the start of the intervention.

The secondary outcome measures are:

• Diabetes self-management (dietary habits, physical activity, monitoring of blood glucose, medication adherence, smoking, GP visits, body mass index (BMI))

• Health-related outcome measures (blood pressure, heart rate, lipid profile, weight, waist and hip circumference)

Intermediate outcome measures are depression, attitude, knowledge, self-efficacy, coping skills, size and composition of participants' social networks, social support, social influence, social engagement

#### Data collection (procedure of effect evaluation and process evaluation)

**Figure 1:**
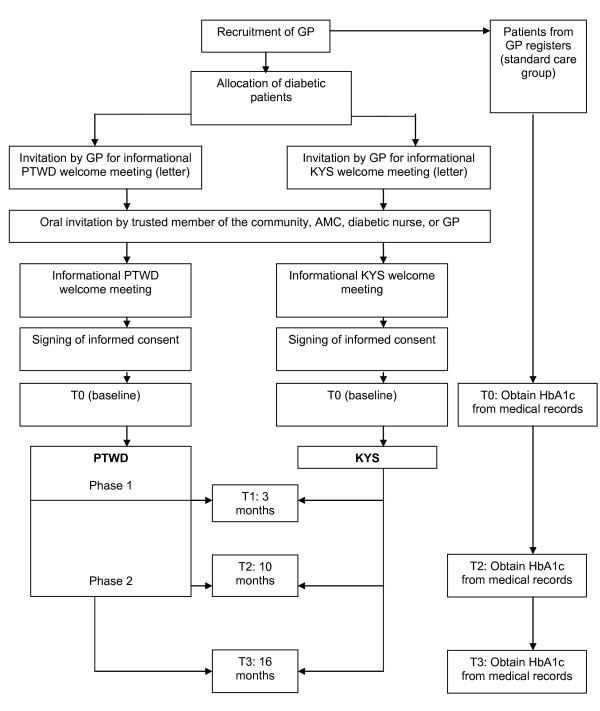
**Measurements in the DISC Study**.

Data collection will take place at baseline (T0), after 3 months (T1), after 10 months (T2), and after 16 months (T3) for both the intervention group (PTWD) and the comparison group (KYS). At T1 we will only administer a short structured interview aimed to measure knowledge and DSM. At T0, T2, and T3 we will administer a structured interview and a physical examination. We will study the participants' medical records at all data collection moments. The HbA1c levels of 128 patients who receive standard care will be collected from a large database owned by a GP collective in the Netherlands. See Figure 1 for an overview of the measurements in this study.

#### Structured interview

Data on diabetes-related behaviour and psychosocial and intermediate outcome measures will be collected during a structured interview, which will be conducted by a trained interviewer. The questionnaire used in these interviews will be translated and back-translated into Turkish by two qualified independent translators and discussed with the researchers. Rather than Arabic, we expect the majority of our Moroccan participants to speak Berber or Moroccan Arabic. Both are spoken languages only. Therefore, the questionnaire will be translated into Berber and Moroccan Arabic during brainstorming sessions with three native speakers and the researchers in which they will reach consensus about the translation. The questionnaire will be written down phonetically'. The female Moroccan and Turkish respondents and the interviewers will be matched according to gender. The interview will take place in the mother tongue of the participant.

As the target population has a low educational level, we searched for questionnaires that fit this level. Hence, we made a selection of existing questionnaires based on their reliability, validity, and inter-rater reliability among diverse ethnic populations and people in lower SE groups. We pretested the Diabetes Problem-Solving Inventory (DPSI), the Diabetes Social Support Questionnaire DSSQ-Friends Version and DSSQ-Family Version, the Theory of Planned Behaviour (TpB) Questionnaire, and the compilation of the Social Capital Question Bank (see Table 2), and adjusted them to optimally match our respondents' ability to answer the questions. We removed items that our target population did not identify with (face validity) or that were irrelevant for this study. Furthermore, a professional adjusted the literacy level of the questionnaires to the lower educational level of our target population, and we adapted some items to make them more culturally sensitive for the different cultural groups in our study population. For example, in the DPSI we adjusted some of the vignettes to make them more appropriate for our different cultural groups. See Table 2 for an overview of the questionnaires we used.

**Table 2:** Overview of the questionnaires used in the DISC Study

Outcome measure	Questionnaire
Diabetes-related quality of life	Diabetes-specific quality-of-life scale [40]

Depression	4DKL (Four-Dimensional Compliant Questionnaire)

Knowledge	SKILLD (spoken knowledge in low literacy in diabetes scale) [41]

Outcome expectations	OEDM-P (Outcome Expectations for Diabetes Self-Management-positive) OEDM-N (Outcome Expectations for Diabetes Self-Management-negative) [42]

Self-efficacy	Diabetes self-efficacy scale [43,44]

Coping skills	DPSI [45]

Social network	Compilation from the Social Capital Question Bank [46,47]

Social support	DSSQ-Family Version DSSQ-Friends Version [48,49]

Social influence	TpB Questionnaire [50]

#### Physical examination

The physical examination in the intervention (PTWD) and comparison group (KYS) will be administered at T0, T2, and T3, and will consist of a standardized measurement of weight, height, waist and hip circumference, blood pressure, and heart rate. An anthropometrical protocol will be used for these measurements. We will monitor the execution of this procedure.

#### Blood and urine samples

In the Netherlands, HbA1c levels and fasting plasma glucose of diabetic patients are measured every three months. An extensive analysis of blood and urine samples is performed at least once a year [39]. For T0, T2, and T3, we will collect these data from the participants' medical records. More specifically, we will collect:

• Fasting plasma glucose, HbA1c, total cholesterol, high-density lipoprotein (HDL) cholesterol, triglycerides, creatinine, and creatinine clearance, glomerular filtration rate calculated according to the Modification of Diet in Renal Disease Study (MDRD equation)

• Microalbuminuria

In addition, we will use medical records to collect information on the participants' medication use and diabetes-related complications: retinopathy, cataract, kidney failure, microalbuminuria, myocardial infarction, angina pectoris, transient ischaemic attack (TIA), cerebrovascular accident (CVA), claudicatio intermittens, diabetic ulcers, amputation, polyneuropathy, and sensitivity problems in the feet.

#### Qualitative study

We will perform a qualitative study to gain in-depth understanding of the key mechanisms of PTWD: changes in the psychosocial mechanisms in the immediate social environments of the participants. We will administer semi-structured qualitative interviews with PTWD and KYS participants. Topics will include overall experiences with the intervention, perceived changes in the immediate social environment (psychosocial mechanisms and social support), and perceived benefits of the intervention regarding coping skills and DSM.

#### Process evaluation

The aim of the process evaluation is to gather in-depth information regarding the fidelity and feasibility of PTWD and KYS. Data collection will take place during the entire intervention period. We will study the journals and files of the group leaders, in which they will record the intervention's implementation and progress. In addition, we will regularly observe group meetings, and we will administer a questionnaire and semi-structured in-depth interviews to the group leaders and the participants.

#### Cost-effectiveness

For both PTWD and KYS, we will calculate the organizational costs (e.g. expenses incurred during the intervention, such as hiring group leaders and locations and developing intervention materials), non-medical costs (e.g. expenses incurred by participants because of their participation in the intervention, such as travel expenses), and medical costs (e.g. medical expenses incurred by participants during the intervention, such as the medication they used and visits to their GPs). We will also compare these costs with an estimation of the expenses incurred by the patients in the standard care group.

### Statistical analyses

#### Assessment of effect

Descriptive statistics will be applied to describe the study population at baseline. To determine the effect of the intervention on HbA1c levels and diabetes-related quality of life and to follow individual change over time, we will use generalized linear mixed models. Potential confounders and effect modifiers (e.g. depression, gender, and ethnicity) will be investigated. We will further examine predictors (including intervention-related predictors) of a decrease in HbA1c levels and an increase in diabetes-related quality of life. If necessary, we will use propensity scores to estimate the effects [51,52]. The level of significance is set at p < 0.05.

The analyses of the qualitative data (semi-structured in-depth interviews with the participants) will be done by two researchers using MAXQDA 10, a programme for qualitative data analysis. We will construct an initial conceptual framework based on the theoretical assumptions of the intervention. The data will be coded according to this framework using an inductive approach to also include other aspects related to the identified themes and concepts. Next, we will sort and synthesize the data using thematic charting and further analyse the data by detecting patterns and developing explanations [53,54].

#### Assessment of process

All qualitative data (journals and files of the group leaders, semi-structured in-depth interviews) will be analysed with MAXQDA according to the principles of content analysis [53]. The quantitative (semi-structured questionnaires) data will be analysed with SPSS using descriptive statistics.

#### Cost-effectiveness

The costs of the interventions will be described and compared to the quality-of-life outcome measures.

## Discussion

Growing evidence suggests that in addition to social support, other psychosocial mechanisms in the immediate social environments of diabetic patients such as social influence and social engagement have a major influence on DSM as well. Diabetic patients in lower SE groups in particular seem to be confronted with strong psychosocial mechanisms that have an adverse effect on their DSM and seem to experience more difficulties in coping with these mechanisms. Therefore, to improve DSM among these groups it seems necessary to target all psychosocial mechanisms in the immediate social environment that impact their DSM: social support, social influence, and social engagement.

PTWD is, to our knowledge, the first culturally sensitive social network intervention targeted to lower SE groups that aims to stimulate the long-term social support of peers and the immediate social environment, and to target existing psychosocial mechanisms through which the immediate social environment negatively influences DSM. The DISC Study evaluates the effectiveness of PTWD.

A limitation of this study might be that blinding the GPs during the study was impossible. Another limitation that might bias the comparability of the groups is that it was impossible to randomize the intervention and comparison groups due to characteristics of the intervention. We think that the chance of selection bias will be small, because both the intervention and the comparison groups will be invited to a lifestyle intervention. We will record all reasons for participation and non-participation among our respondents, which will enable us to assess any selection bias. In addition, we will control for differences between the groups at baseline and take these differences into account during the analyses.

The present study will be strengthened by the use of data triangulation. We will use a wide variety of sources of information such as questionnaires, semi-structured qualitative interviews, medical records, physical examinations, and the journals and files of the group leaders. This will increase the validity of this study. Moreover, the qualitative semi-structured interviews will reveal in-depth information about lower SE groups that is lacking to date.

This study will provide insights into how to enable diabetic patients in lower SE groups to optimally manage their diabetes by intervening in the psychosocial mechanisms in the social environment that negatively impact their DSM: social support, social influence, and social engagement. If PTWD is effective in lowering the HbA1c levels of the participants and improving their diabetes-related quality of life, further implementation will be considered. PTWD could be implemented in the context of a GP practice, where it could contribute to more efficient diabetes care for diabetic patients in lower SE groups.

## Abbreviations

DISC: Diabetes in social context study; SE: Socioeconomic; DSM: Diabetes self-management; PTWD: *Powerful Together with Diabetes*; KYS: *Know your Sugar*; NIGZ: Dutch national institute for health promotion and disease prevention; GP: General practitioner; DPSI: Diabetes problem-solving interview; DSSQ: Diabetes social support questionnaire; TpB: Theory of planned behaviour; BMI: Body mass index; HbA1c: Haemoglobin A1C; SO: Significant others; AMC: Academic medical center; TIA: Transient ischaemic attack; CVA: Cerebrovascular accident.

## Competing interests

The authors declare that they have no competing interests.

## Authors' contributions

CV coordinates the study, and developed the intervention, constructed the design, and drafted the manuscript. DG developed the outline of the intervention and the intervention materials. VN and KS developed the study, constructed the design, and revised the manuscript. PJMU, BJCM, and GN participated in the design of the study and revised the manuscript. All authors read and approved the final manuscript.

## Pre-publication history

The pre-publication history for this paper can be accessed here:

http://www.biomedcentral.com/1471-2458/12/199/prepub

## Supplementary Material

Additional file 1 **Summary of behavioural goals, determinants addressed, and strategies used in *PTWD *phase 1**. Click here for file

Additional file 2 **Summary of behavioural goals, determinants addressed, and strategies used in *PTWD *phase 2**. Click here for file

Additional file 3 **Summary of behavioural goals, determinants addressed, and strategies used in the meetings for significant others of *PTWD***. Click here for file

Additional file 4 **Summary of behavioural goals, determinants addressed, and strategies used in the social network meetings of *PTWD***. Click here for file
