# Supplementary material for: The DISC (Diabetes in Social Context) Study-evaluation of a culturally sensitive social network intervention for diabetic patients in lower socioeconomic groups: a study protocol
Source: BMC Public Health. 2012 Mar 19;12:199. doi: 10.1186/1471-2458-12-199 (PMC3337803; doi:10.1186/1471-2458-12-199)
Supplement: Additional file 1 — Summary of behavioural goals, determinants addressed, and strategies used in PTWD phase 1. [file 1471-2458-12-199-S1.PDF]

Additional file 1. Summary of behavioural goals, determinants addressed, and strategies used in *PTWD* phase 1

	**Behavioural goals**	**Main determinants addressed**	**Strategies (programme components)**
Phase 1<br>(1-3 months)	Knowing what diabetes is and how to optimally manage it (monitoring of blood glucose, medication adherence, diet, physical activity)	Knowledge	Interactive diabetes quiz<br>Repetition of information
	Feeling that DSM is necessary and beneficial	Outcome expectations	Positive role-model stories<br>Shared positive experiences from group members (vicarious reinforcement)<br>Group discussions
	Believing that you are the one who should and can influence the course of your diabetes	Moral norms	Positive role-model stories<br>Shared positive experiences from group members (vicarious reinforcement)<br>Group discussions
		Self-efficacy	Letter of the week<br>Rehearsal situations<br>Real-life ‘excursions’
	Asking for social support in having and dealing with diabetes	Social support	Interactive games (self-affirmation)<br>Stimulating the exchange of advice<br>Positive role models

			focused on social support<br>Group meetings for significant others
	Being aware of the effect psychosocial mechanisms have on your DSM and being able to deal with these mechanisms	Skills	Letter of the week<br>Rehearsal situations
		Social influence<br>Perceived cultural norms	Role-model stories<br>Letters of the week<br>Rehearsal situations<br>Group discussions
**Selection of behavioural goals regarding social support in Phase 1**	The participants trust each other and share experiences with each other	Outcome expectations<br>Perceived norms<br>Moral norms<br>Self-efficacy and skills<br>Social support<br>Social influence	Setting rules for the disclosure of information in the group (no gossiping)<br>Interactive games and energizers to build trust: giving each other compliments, weaving the web of life, solving puzzles together, sharing positive news at home with each other
	Participants listen carefully to each other and give each other positive feedback	Outcome expectations<br>Perceived norms<br>Moral norms<br>Self-efficacy and skills<br>Social support<br>Social influence	Communication skills (giving positive feedback)<br>Focus of group leader on listening and positive feedback<br>Practice with guided feedback

DSM: diabetes self-management
