# Supplementary material for: The DISC (Diabetes in Social Context) Study-evaluation of a culturally sensitive social network intervention for diabetic patients in lower socioeconomic groups: a study protocol
Source: BMC Public Health. 2012 Mar 19;12:199. doi: 10.1186/1471-2458-12-199 (PMC3337803; doi:10.1186/1471-2458-12-199)
Supplement: Additional file 2 — Summary of behavioural goals, determinants addressed, and strategies used in PTWD phase 2. [file 1471-2458-12-199-S2.PDF]

Additional file 2. Summary of behavioural goals, determinants addressed, and strategies used in *PTWD* phase 2

	**Behavioural goals**	**Main determinants addressed**	**Strategies (programme components)**
Phase 2 (4-12 months)	Identifying problems and barriers to your DSM (monitoring of blood glucose, medication adherence, diet, physical activity)	Awareness of barriers	Diaries<br>Role-model stories<br>Exchange of experiences
	Being able to come up with possible solutions for these barriers	Problem-solving skills	Participatory problem-solving<br>Rehearsal situations<br>Homework assignments followed by feedback
		Self-efficacy	Rehearsal situations<br>Homework assignments followed by feedback
	Actively involving family and/or friends when overcoming these barriers	Communication strategies	Role-model stories focused on these topics<br>Rehearsal situations focused on these topics<br>Homework assignment focused on these topics
		Self-efficacy	
		Asking for and receiving social support	

		Handling social influence on DSM	Encouraging the participants to see each other in between the group meetings and undertake DSM activities (e.g. exercising) together<br>Social network meetings with significant others
	Being able to evaluate and improve the strategies chosen to overcome these barriers	Self-efficacy	Rehearsal situations<br>Homework assignments followed by feedback
		Problem-solving skills	Participatory problem-solving<br>Rehearsal situations<br>Homework assignments followed by feedback
**Selection of behavioural goals regarding social support in Phase 2**	The participants feel like a team	Outcome expectations<br>Perceived norms<br>Moral norms<br>Collective self-efficacy and skills<br>Social support<br>Social influence<br>Social engagement	Focus of group leader on togetherness and supporting each other<br>Participatory problem-solving<br>Team-building energizers
	The participants continue to support each other outside of the group meetings	Outcome expectations<br>Perceived norms<br>Moral norms<br>Collective self-efficacy	Focus of group leader on the continued existence of the group<br>Guided practice with

		and skills<br><br>Social support<br><br>Social influence	feedback<br><br>Making appointments with other group members

DSM: diabetes self-management
