# Supplementary material for: The DISC (Diabetes in Social Context) Study-evaluation of a culturally sensitive social network intervention for diabetic patients in lower socioeconomic groups: a study protocol
Source: BMC Public Health. 2012 Mar 19;12:199. doi: 10.1186/1471-2458-12-199 (PMC3337803; doi:10.1186/1471-2458-12-199)
Supplement: Additional file 3 — Summary of behavioural goals, determinants addressed, and strategies used in the meetings for significant others of PTWD. [file 1471-2458-12-199-S3.PDF]

Additional file 3. Summary of behavioural goals, determinants addressed, and strategies used in the meetings for significant others of *PTWD*

**Behavioural goals**	**Main determinants addressed**	**Strategies (programme components)**
SOs know what diabetes is and how to optimally manage it (monitoring of blood glucose, medication adherence, diet, physical activity)	Knowledge	Interactive diabetes quiz<br>Repetition of information
SOs believe that the DSM of the diabetic patient is a shared responsibility	Moral norms and outcome expectations	Positive role-model stories<br>Shared positive experiences of other SOs (vicarious reinforcement)<br>Group discussions
SOs regularly inquire how things are going with the management of diabetes (monitoring of blood glucose, medication adherence, diet, physical activity)	Social influences<br>Perceived norms and social expectations	Role-model stories<br>Letter of the week<br>Rehearsal situations<br>Group discussions
	Moral norms and outcome expectations	Positive role-model stories<br>Shared positive experiences of other SOs (vicarious reinforcement)<br>Group discussions
	Self-efficacy and (communication) skills	Letter of the week<br>Rehearsal situations

If the DSM is going well, the SO gives compliments to the diabetic patient<br><br>If the diabetic patient is experiencing difficulties with his/her DSM, the SO gives positive feedback	Outcome expectations	Positive role-model stories<br>Shared positive experiences from group members (vicarious reinforcement)<br>Group discussions
	Self-efficacy and (communication) skills	Letter of the week<br>Rehearsal situations
	Perceived norms and social expectations	Role-model stories<br>Letter of the week<br>Rehearsal situations<br>Group discussions

DSM: diabetes self-management

SO: significant other
