# Supplementary material for: The DISC (Diabetes in Social Context) Study-evaluation of a culturally sensitive social network intervention for diabetic patients in lower socioeconomic groups: a study protocol
Source: BMC Public Health. 2012 Mar 19;12:199. doi: 10.1186/1471-2458-12-199 (PMC3337803; doi:10.1186/1471-2458-12-199)
Supplement: Additional file 4 — Summary of behavioural goals, determinants addressed, and strategies used in the social network meetings of PTWD. [file 1471-2458-12-199-S4.PDF]

Additional file 4. Summary of behavioural goals, determinants addressed, and strategies used in the social network meetings of *PTWD*

**Behavioural goals**	**Determinants addressed**	**Strategies (programme components)**
SO and diabetic patient together identify the diabetic patient's obstacles for DSM (monitoring of blood glucose, medication adherence, diet, physical activity)	Awareness of barriers	Exchange of experiences and thoughts on DSM<br>Discussion of the diabetic patient's personal action plan
SO and diabetic patient together make a plan to overcome these obstacles	Problem-solving skills	Group discussion<br>Participatory problem-solving<br>Guided feedback from group leader
SO and diabetic patient execute the plan they made together	Collective self-efficacy	Homework assignment followed by feedback
SO and diabetic patient evaluate the plan and make adjustments if necessary	Collective self-efficacy	Giving each other positive feedback<br>Guided feedback from group leader
	Problem-solving skills	Group discussion<br>Participatory problem-solving<br>Guided feedback from group leader

DSM: diabetes self-management

SO: significant other
